# Evolution of Fungal Carbohydrate-Active Enzyme Portfolios and Adaptation to Plant Cell-Wall Polymers

**DOI:** 10.3390/jof7030185

**Published:** 2021-03-05

**Authors:** Hayat Hage, Marie-Noëlle Rosso

**Affiliations:** INRAE, Aix Marseille University, UMR1163, Biodiversité et Biotechnologie Fongiques, F-13009 Marseille, France; hayat.hage@etu.univ-amu.fr

**Keywords:** CAZyme, fungal ecology, evolution, plant-cell wall, plant biomass

## Abstract

The postindustrial era is currently facing two ecological challenges. First, the rise in global temperature, mostly caused by the accumulation of carbon dioxide (CO_2_) in the atmosphere, and second, the inability of the environment to absorb the waste of human activities. Fungi are valuable levers for both a reduction in CO_2_ emissions, and the improvement of a circular economy with the optimized valorization of plant waste and biomass. Soil fungi may promote plant growth and thereby increase CO_2_ assimilation via photosynthesis or, conversely, they may prompt the decomposition of dead organic matter, and thereby contribute to CO_2_ emissions. The strategies that fungi use to cope with plant-cell-wall polymers and access the saccharides that they use as a carbon source largely rely on the secretion of carbohydrate-active enzymes (CAZymes). In the past few years, comparative genomics and phylogenomics coupled with the functional characterization of CAZymes significantly improved the understanding of their evolution in fungal genomes, providing a framework for the design of nature-inspired enzymatic catalysts. Here, we provide an overview of the diversity of CAZyme enzymatic systems employed by fungi that exhibit different substrate preferences, different ecologies, or belong to different taxonomical groups for lignocellulose degradation.

## 1. Introduction

The massive population growth that the world faces today is causing an increase in greenhouse gas emissions, of which carbon dioxide (CO_2_), methane (CH_4_), nitrous oxide (N_2_O), and fluorinated gases (F-gases) are the most significant. Strikingly, CO_2_ concentration in the atmosphere increased by 47% following the Second Industrial Revolution in the early 1900s [1]. As a result, human-induced climate warming is now estimated to rise above +1.5 °C by 2050 [2]. This excess of CO_2_ originates primarily from fossil fuels that are burned to fulfil human energy needs. Scientists and environmentalists warn that, if nothing is done to address global warming, catastrophic consequences may occur on the social and economic levels, and ecosystems will be unable to naturally adapt to changes in the climate. In some scenarios, stabilizing the climate requires drastic reductions in energy demand while overall living standards rise. Such scenarios are dependent on technological innovation for the rapid decarbonization of energy supply, low-carbon technology innovation, and well-managed land systems that result in a net uptake of CO_2_ from the atmosphere via, for example, afforestation, reforestation, land restoration, and soil carbon sequestration. Globally, it is estimated that 477 Pg of carbon (C) are stored in forest soils, litter, and deadwood [3]. Soil microbes associated with the rhizosphere, litter, or deadwood impact the balance of carbon storage vs. CO_2_ emissions. Symbiotic fungi may promote plant growth, and thereby increase CO_2_ assimilation via photosynthesis. Conversely, litter and deadwood decayers may prompt the decomposition of dead organic matter, and thereby the release of organic compounds that can be assimilated by other organisms and contribute to CO_2_ emissions via respiration [4]. It is estimated that wood-decay basidiomycetes decompose 120 ton/km^2^ of wood each year, resulting in CO_2_ efflux [5]. The dynamics of deadwood decay are related to successions of fungal communities and the ability of wood decayers to use different components of lignocellulosic materials as a carbon source [6,7,8,9,10]. For example, white-rot fungi can mineralize lignin with the ultimate formation of CO_2_ and H_2_O [11,12]. The pivotal role that white-rot fungi play in forest ecosystems stimulated research efforts to understand the enzymatic mechanisms involved in wood degradation.

Another challenge for sustainability over time is the limited capability of the physical environment to absorb the waste of human activities. Most commodity chemicals are currently produced from platform molecules obtained from fossil fuels. These platform molecules are produced to meet diverse industrial needs in the manufacture of plastics, synthetic fibers, synthetic rubber, dyes, pigments, paints, coatings, fertilizers, agricultural chemicals, pesticides, cosmetics, soaps, detergents, and pharmaceuticals. New technologies must be developed to meet human needs for chemical products using renewable resources in place of persistent, bioaccumulative, toxic, and otherwise hazardous materials. Plant biomass is considered to be a clean resource since approximately the same amount of carbon is emitted during its conversion as what is absorbed during the photosynthetic process, thus preserving the carbon balance. However, the protection of available land and forest against deforestation, urbanization, and desertification shapes the potential of biomass in energy and organic chemical production. In addition, the use of plant biomass should not compete with land use for agriculture or food and feed production. Thus, it is necessary to use nonedible residue and waste from forestry, agriculture, or agroindustry as renewable resources. Another challenge for sustainable biorefineries is the use of local biomass feedstocks to suppress the energy cost of long-distance transport. The combination of these criteria implies that the remaining biomass feedstock is highly diverse and recalcitrant to degradation (e.g., straws, wood sawdust, and seed-oil press cakes). The conversion of such lignocellulosic materials is limited by the complexity of the lignocellulosic structure. In particular, the highly recalcitrant lignin polymer and the crystalline regions of cellulose microfibrils limit the breakdown process and make it energy-consuming [13]. Thus, in a biobased economy, efforts are made to find environmentally friendly and economically sustainable methods for the conversion of biomass polymers in value-added molecules and fibers. One solution lies in microorganisms that possess abilities to efficiently break down plant-cell-wall (PCW) polymers. Of these microorganisms, fungi are the predominant source of enzymes currently used on an industrial scale for biomass transformation [14].

During evolution, fungi that use plant tissue as a carbon source acquired a large diversity of PCW-degrading or -modifying enzymes (PCWDE; Table 1). These enzymes are classified with all carbohydrate-active enzymes (CAZymes) identified to date into families in the CAZy database (http://www.cazy.org/ [15], accessed on 23 January 2021) according to their amino acid sequence and structural similarity. A comparison of the repertoires of genes coding for CAZymes (CAZomes) in the genomes of fungi from different taxa, having different ecologies or different substrate preferences, is critical to strengthening our understanding of the role that fungi play in the carbon balance of soils. In addition, the discovery of new enzymes that degrade PCW polysaccharides is essential to improve the enzymatic cocktails aimed at plant-biomass transformation in industrial processes. 

In this review, we outline the current understanding of how evolution shaped fungal CAZomes in regards to their adaptation to diverse habitats and lifestyles. We review recent findings on the enzymatic systems used by fungi for lignocellulose degradation, with an emphasis on wood-decay fungi from the order of Polyporales, which remains a focus of research efforts in recent years.

## 2. Fungal Adaptations to Land Plants Paralleled by Evolution of Fungal CAZomes

**Figure 1:**
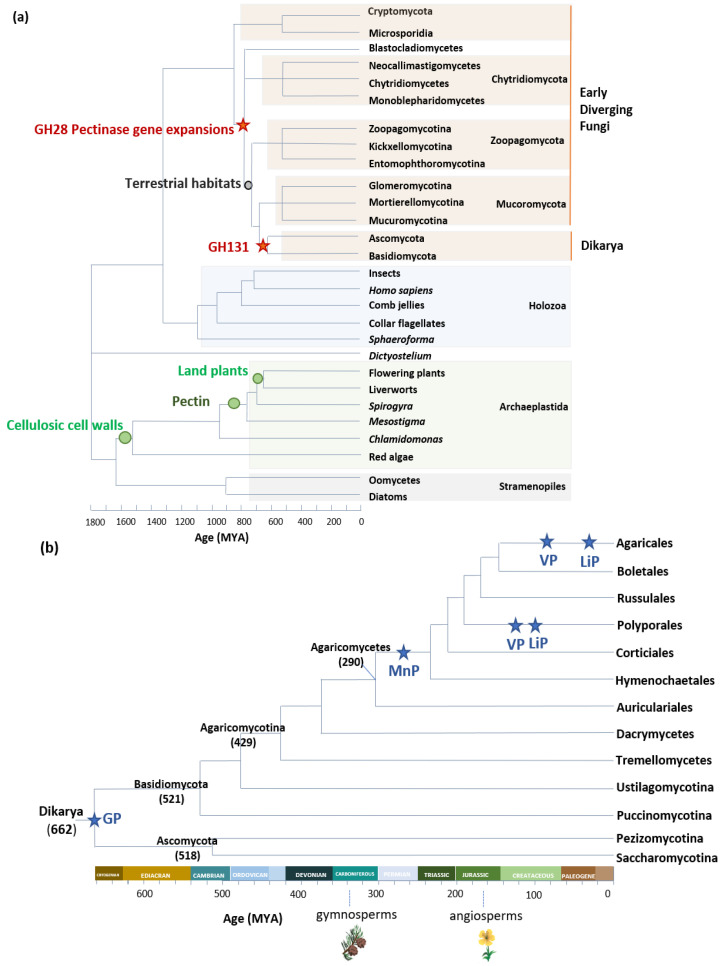
Early evolutionary events for (**a**) GH28 and GH131 enzymes, and (**b**) Class II peroxidases. Estimated ages of fungal taxa from Floudas et al., Chang et al., Berbee et al., Spatafora et al., Ayuso-Fernández et al., and Ruiz-Dueñas et al. [30,37,38,39,40,41].

It is estimated that fungus–plant associations originated around 750 million years ago, the approximate time of the emergence of pectin-containing streptophyte algae [24,25,26]. The cell walls of streptophyte algae contained cellulose, xyloglucans, and pectin, but lacked lignin [27]. On their side, early diverging fungi that lived in association with streptophytes possessed cellulase, xyloglucanase, and pectinase genes in their genome, indicating that these enzymes were part of the ancestral fungal toolkit for breaking down plant material (Figure 1a) [24,25,26,28]. Later, pectinase genes were convergently lost in lineages that adopted a nonstreptophyte nutrition, whereas organisms that had adopted a plant-based nutrition were under continuous selection pressure to retain pectinase genes [29]. Furthermore, in some lineages, pectinase genes underwent rapid duplications [30]. In a recent study, Anasontzis et al. (2019) analyzed the distribution and enzymatic activity of GH131, a CAZy family only found in fungi and in plant-parasitic oomycetes. The authors gathered an array of indications that GH131 could have contributed to fungal adaptation to land-plant tissue. First, they showed that GH131 appeared in a common ancestor of Dikarya, a subkingdom of fungi that arose around 662 million years ago (Mya) [31], shortly after land plants (703 ± 45 Mya [32]). Gene copy numbers were higher in extant species of fungi that live on plant tissue, i.e., plant symbionts, plant pathogens, and saprophytic fungi, than in animal or fungal pathogens. GH131 have endo-β-1,3 and endo-β-1,4 glucanase activity, and can thereby cleave the mixed β-1,3/1,4 glucan bonds of hemicelluloses and the β-1,4 glucan polymers of cellulose. Some of the copies have the catalytic domain fused to a CBM1, a carbohydrate-binding module that has an affinity to crystalline cellulose, and directs CBM1-associated enzymes to potentiate cellulolytic activities on insoluble substrates [33,34]. The transcription of GH131 genes from symbiotic, pathogenic, and saprophytic fungi was upregulated during plant-tissue colonization, and GH131 genes shared the same transcription profile as genes coding for well-known cellulose-degrading CAZymes. Lastly, the cosecretion of GH131 with AA9 lytic polysaccharide monooxygenases (LPMOs), cellobiohydrolases, and other endoglucanases [35] supported the fact that GH131 act in synergy with other enzymes for PCW remodeling and degradation, and contributed to the adaptation of fungi to growth in the tissue of land plants.

Another example of fungal adaptation to land plants is the ability to degrade lignin. This ability parallels the evolution of lignin-active class II peroxidases (POD) in Agaricomycetes, a class of fungi among Basidiomycota. POD are classified into four groups: generic peroxidase (GP), a group of nonligninolytic low-redox potential peroxidases, and three ligninolytic forms—manganese peroxidase (MnP), versatile peroxidase (VP), and lignin peroxidase (LiP). Using phylogenomics, Floudas et al. [31] demonstrated that the most recent common ancestor of Agaricomycetes contained the gene coding for nonligninolytic GP. The acquisition of an Mn(II)-binding site, formed by two glutamate and one aspartate residues, allowed for the novel enzymes (MnP) to catalyze the oxidation of lignin phenolic groups via Mn^3+^ chelates [36]. The estimated period of this transition from nonligninolytic GP to ligninolytic MnP was the late Carboniferous period, after the accumulation of lignin-containing biomass on Earth [31]. In some lineages, the catalytic properties of POD further evolved in parallel with the evolution of lignin complexity in plants. The modification of lignin composition in angiosperms drove, in some fungal lineages, the evolution of MnP toward VP, in which the appearance of a surface tryptophan residue conferred to the enzyme the ability to abstract electrons from nonphenolic groups and to transfer them to the hemic cofactor via a long-range electron transfer (LRET) [37]. Lastly, LiPs appeared in the Polyporales and Agaricales taxa by convergent evolution from VP ancestor genes after the loss of the Mn(II) binding site (Figure 1b) [37]. In a recent phylogenomic study on 33 Agaricales genomes, F.J. Ruiz-Dueñas et al. further investigated the occurrence of sequence polymorphism in the Mn(II) binding site of MnP and VP enzymes, and proposed that the observed amino acid substitutions could “be the result of exploration of new mechanisms to modify lignin” in fungi living in diverse ecological niches (buried wood, decayed wood, leaf litter, and grass litter) [38].

## 3. CAZomes of Saprophytic Fungi

### 3.1. Wood-Decay Fungi

**Figure 2:**
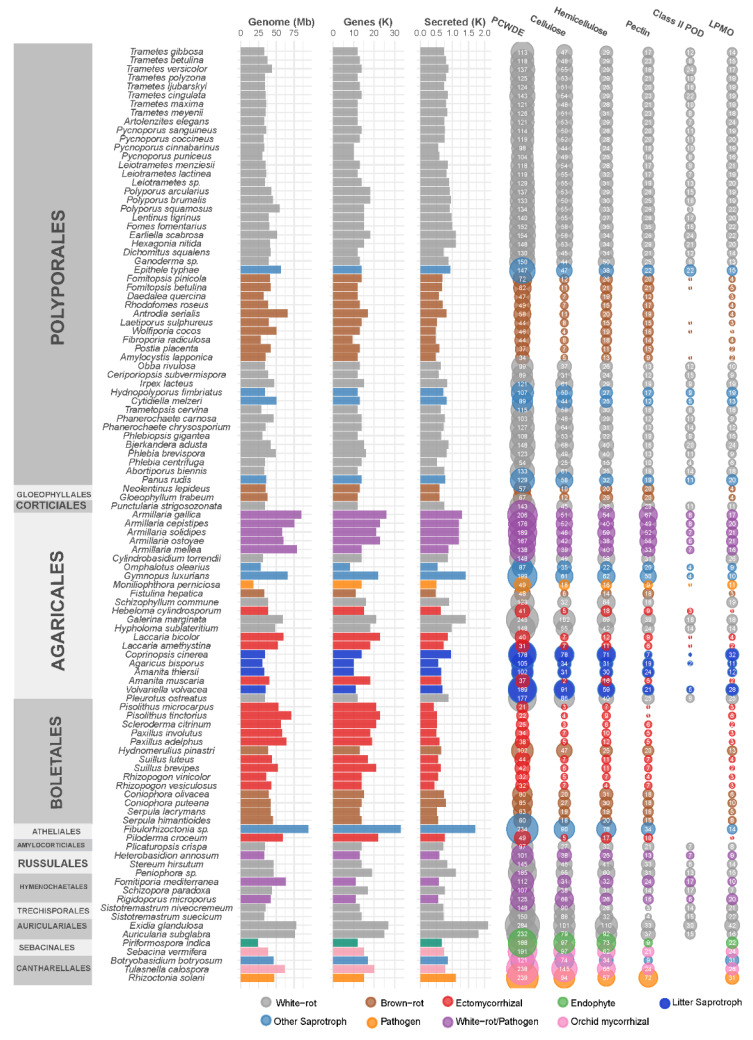
Genomic features of 107 Agaricomycetes and gene counts for secreted enzymes active on cellulose, hemicellulose, or pectin, and for Class II peroxidases and lytic polysaccharide monooxygenases (LPMOs) AA9, AA10, AA13, and AA14.

Among saprobes, wood-decay fungi can be classified into three major types on the basis of the aspect of the wood following fungal colonization: white-, brown-, and soft-rot fungi. White-rotters, the dominant wood-degrading species, are named as such due to the white and fibrous decayed wood that they produce. They are rich in PCWDE and can degrade the entirety of the PCW polymers, including cellulose, hemicellulose, pectin, and lignin. In contrast, the wood decayed by brown-rot fungi shows dry and brown cubic debris in which cellulosic and hemicellulosic compounds are degraded, whereas lignin is oxidized and only partially degraded. These fungi have reduced gene portfolios for PCWDE, and produce reactive hydroxyl radicals via the Fenton reaction to cleave PCW polymers [42,43,44]. Soft-rot fungi typically attack wood with reduced lignin content in high-moisture conditions, and can cause the grayish discoloration and fragmentation of the decayed wood. Although this classification facilitates comparative analyses of wood-decay types, the white-/brown-rot dichotomy tends to not be representative of the diversity of fungal degradation strategies [45]. Indeed, the presence of ligninolytic POD genes in a genome was first correlated to the classification of the species as a white-rotter [31,46,47]. However, some species produce a white-rot phenotype despite lacking ligninolytic POD in their genome [19,47]. These fungi could be in transition between white- and brown-rot decay types, similar to transitions that took place independently at least three times during the evolution of Agaricomycetes [48]. Typical white- and brown-rot fungi deploy a characteristic panel of enzymatic and chemical mechanisms for the degradation of PCW polymers (reviewed in Lundell et al. [49]). Brown-rot fungi possess a significantly smaller set of PCWDE than that of white-rot fungi (Figure 2). Notably, CAZymes related to the depolymerization of crystalline cellulose (i.e., lytic polysaccharide monooxygenases (LPMOs) from CAZy family AA9) or lignin (i.e., POD, multicopper oxidases MCO and H_2_O_2_-generating GMC oxidoreductases) are expanded in white-rot fungi as compared to brown-rot fungi [31,46]. Brown-rot fungi show peculiar gene expression patterns, and the induction of ferric reductase and hemethiolate peroxidase/peroxygenase genes in the early stages of wood degradation [50].

### 3.2. Polyporales

**Figure 3:**
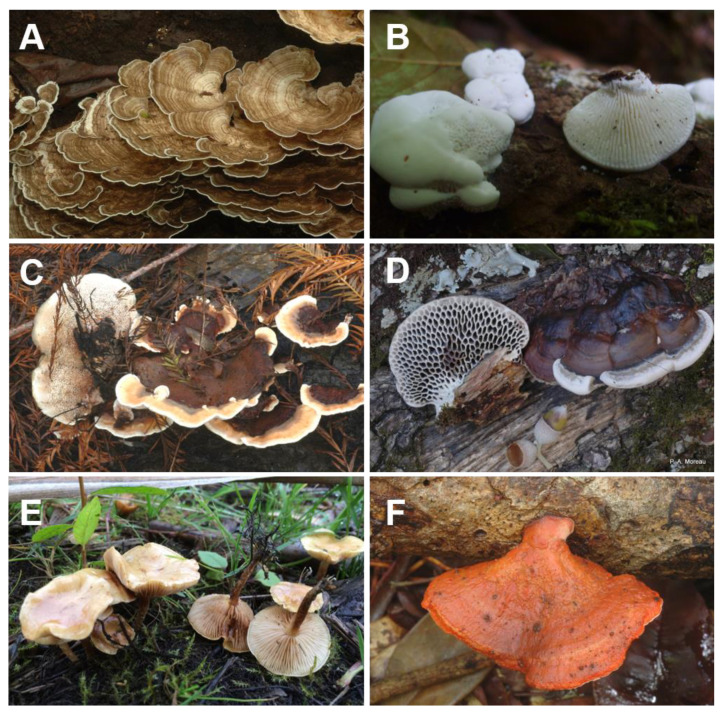
Examples of Polyporales fungi. (**A**) *Leiotrametes menziesii*, (**B**) *Artolenzites elegans*, (**C**) *Abortiporus biennis*, (**D**) *Hexagonia nitida*, (**E**) *Pholiota conissans*, (**F**) *Pycnoporus puniceus*. Pictures kindly provided by Cony de Cock (Université Catholique de Louvain, Belgium), Stéphane Welti (Université de Lille, France), Pierre-Arthur Moreau (Université de Lille, France), and Régis Courtecuisse (Université de Lille, France).

Among wood-decay fungi, species from the order of Polyporales are of great interest due to their remarkable efficiency in wood degradation (Figure 3). Phylum-wide phylogenomic studies indicated that high diversification rates occurred in Polyporales [51], which possibly shaped a diversity of enzymatic mechanisms to adapt to diverse ecological niches and substrates. This was recently supported by the observation of different evolutionary trajectories for PCWDE gene portfolios among Polyporales [52]. Notably, recent genome-sequencing efforts assisted in the discovery of new families of LPMOs that cleave crystalline cellulose [23] or the xylan polymers coating cellulose microfibrils [21]. Some enzymes that indirectly contribute to PCW degradation were also first characterized in Polyporales, such as aryl-alcohol dehydrogenases from white-rot fungus *Pycnoporus cinnabarinus* [53].

Among white-rot Polyporales, different species can degrade lignin with different degrees of cellulose preservation. This diversity in degradation abilities was even observed between species from the same genus. For example, *Phanerochaete chrysosporium* appeared to simultaneously degrade lignin along with all cell-wall carbohydrates, whereas *Phanerochaete carnosa* showed selective (i.e., predominant) removal of lignin with mostly no loss of cellulose [54]. The selective degradation of lignin is interesting for biorefineries aimed at the transformation of cellulose and hemicelluloses, as the removal of the lignin barrier improves yields in saccharide recovery. As an example, fungal strain *Polyporus brumalis* BRFM 985 was selected from a screen of 63 white-rot fungal strains for the selective delignification of wheat straw under solid-state fermentation [55]. Genome analysis showed the expansion of the POD gene family (19 genes), including MnP and VP, and of GMC oxidoreductases/dehydrogenases (36 genes). GMC oxidoreductases/dehydrogenases assist in lignin breakdown by generating H_2_O_2_ or by reducing the oxidation products of lignin. Strikingly, the fungus secreted an unprecedented number of POD during fermentation (5 MnP and 6 VP), which confirmed the ability of *Polyporus brumalis* to efficiently drive oxidative machinery to break down lignin [56]. Furthermore, different Polyporales species use different sets of enzymes to degrade lignin. For example, the numbers of lignin-active POD vary significantly from one species to another. Indeed, no POD from the VP family was identified in the genome of *Phanerochaete chrysosporium*, whereas nine genes were found in that of *Polyporus brumalis*. Similarly, some species (within the phlebioid clade) have no laccase genes in their genome (e.g., *Phanerochaete chrysosporium*, *Phanerochaete carnosa* [54]), whereas four and five laccase genes were identified in *Phlebia centrifuga* and *Phlebia radiata*, respectively [57]. Such high diversity in enzymatic systems and enzyme–gene repertoires stimulates the screening of the natural biodiversity within Polyporales for the identification of the most efficient strains and enzyme sets aimed at plant-biomass valorization [55,58].

### 3.3. Litter-Decay Fungi

Among saprotrophic fungi, some litter-decomposing species in forests and grasslands are notably rich in enzymes acting on the lignin and polysaccharide fractions of the PCW. For example, litter-decomposing species from the order of Agaricales have significantly higher gene copy numbers for PCWDE appended to a CBM1, for laccase, and AA9 LPMO (Figure 2). CBM1-appended catalytic domains could prevent enzyme leaching in a loose litter environment as compared with compact wood. In addition, the adaptation of Agaricales to diverse ecological niches (e.g., forest litter, grass litter, deadwood, buried wood) parallels a surprisingly high-protein sequence diversification in ligninolytic peroxidases, which evolved from an ancestral MnP isoform to MnP, VP, and LiP isoforms, with diverse protein lengths and amino acids engaged at the catalytic site [38].

## 4. CAZomes of Mycorrhizal Fungi

Mycorrhizal fungi establish a mutualistic association with their host. They colonize the plant to extract the required carbon for their nutrition, while the plant benefits from the water, phosphorus, and nitrogen conveyed from the soil by the fungus [59]. To establish this association, mycorrhizal fungi developed different strategies to colonize plant tissue over more than 500 million years of coevolution with plants. Mycorrhizal fungi are classified into four main types according to the morphological diversity of the symbiotic structures. Arbuscular mycorrhizae (AM) are formed by Glomeromycetes that develop arbuscules inside the root cells of the host. Ectomycorrhizae (ECM) are formed by higher fungi that produce a labyrinthine hypha between host cells. Orchid mycorrhizae fungi (ORM) form coils of hyphae (pelotons) that penetrate the cells of Orchidaceae plants. Lastly, ericoid mycorrhizae (ERM) are formed by some ascomycete fungi and characterized by pelotons entering the very thin roots of Ericaceae [60].

ECM fungi have a reduced set of CAZymes as compared to those of saprotrophic fungi, which enable the tuned remodeling of the PCW for nutrient exchange with minimal disruption. Their enzymatic portfolio is similar to that of brown-rot fungi [61,62], both having lost most of the plesiomorphic enzymatic machineries of their white-rot ancestors (Figure 2). For example, cellulases GH6 and GH7 (cellobiohydrolase), hemicellulase CE1 (acetylxylan esterase), pectate lyases, and lignin-active POD are absent in ECM species [36,47]. Conversely, GH5_5 (β-1,4-endoglucanase), GH12 (β-1,4-endoglucanase), and AA9 LPMO, which target cellulose, and GH28 (polygalacturonase), which targets pectin, could play a role in the early steps of the colonization of plant rootlets during the penetration of the plant root cortex [63]. An elegant demonstration of the role of GH5_5 in this process was provided by the reduced numbers of mycorrhizae formed by *Laccaria bicolor* when the GH5_5 gene was knocked down [64]. Of note, despite overall gene loss for PCW-degrading enzymes, mycorrhizal genomes seemed to undergo expansions of laccase genes. These laccases could be related to fruiting body development [39].

Genome sequencing efforts show that AM fungi also have reduced portfolios for PCWDE genes. Strikingly, the opposite was found in the genomes of ERM, ORM and endophyte fungi, which are rich in PCWDE genes. These unexpected CAZomes are proposed to mirror a versatile lifestyle of the fungi, and their ability to behave as saprotrophs in the absence of a plant host [65].

## 5. Differences in CAZyme Gene Expression Profiles Contribute to Functional Diversity among Fungi

Beyond gene portfolios for PCWDE, functional diversity may arise from the regulation of gene expression on the transcriptional, translational, or post-translational level. 

In filamentous fungi, the production of PCWDE is largely regulated by the availability and abundance of carbon resources. In several ascomycete fungi, cellulase and hemicellulase production is repressed when the fungi are in the presence of easily assimilable mono- or disaccharides through carbon catabolism repression (CCR). On the other hand, the limitation of nutrient resources causes an increase in the expression of ligninolytic POD genes [66]. In addition, other environmental parameters such as temperature, pH, and the presence of ions or reactive oxygen species contribute to the regulation of the expression of PCWDE genes [67]. Recently, CCR was shown to occur in Basidiomycetes *Pleurotus ostreatus* and *Dichomitus squalens* [66,68]. However, despite efforts to identify the transcription factors involved in CCR in Basidiomycota [69], they are still largely unknown.

Several transcriptomic studies showed that the transcription of PCWDE genes is finely regulated in Basidiomycetes during growth on biomass feedstocks. For example, in white-rot fungus *Pycnoporus coccineus*, a set of CAZymes were simultaneously expressed and secreted 3 days after transfer from a culture medium containing maltose to a medium containing lignocellulose as the sole carbon source [66]. Among these enzymes, some targeted cellulose and β-1,4-glycans (AA9 LPMO, GH3, GH5, GH6, GH7, GH45, and GH131). Others targeted hemicellulose: GH10, which cleave the main chain of xylan; GH43 and GH51 arabinofuranosidases, which cleave the arabinose substitution of xylan; GH115 glucuronidases, which cleave the glucuronoyl substitution of woody xylan; CE4 and CE16, which target acetyl xylans; and CE15, which the target 4-O-methyl glucuronoyl side chains of hemicellulose [70]. The set of induced CAZyme genes was enriched in enzymes fused to a CBM1 module. Noticeably, eight AA9 LPMO genes were strongly upregulated in a transcription profile that resembled that of the cellobiose dehydrogenase (CDH) gene, which suggested that CDH might promote the activity of these LPMOs via electron transfer, as suggested from in vitro enzymatic assays [71]. The transcription of this set of genes further increased after 7 day growth on the lignocellulosic substrates. Conversely, the transcript level of lignin-active POD decreased at Day 7. A delay in the secretion of POD enzymes was noted, as observed in white-rot fungi *Phanerochaete carnosa* and *Ceriporiopsis subvermispora* [72,73]. The tight regulation of the expression of POD might allow for the deconstruction of lignin polymers, and facilitate the access of cellulolytic and hemicellulolytic enzymes to their substrates while preventing damage to the hyphae by oxidized compounds. The tuned regulation of the expression of PCWDE was also elegantly shown on miniaturized wood-colonization assays with brown-rot fungus *Postia placenta* [74]. The authors observed reactive oxygen species (ROS) generation at the hyphal front, whereas hydrolytic enzymes were produced at later stages of the hyphal progression in the wood. Uncoupling oxidative degradation of lignin from the hydrolytic degradation of polysaccharides likely resulted in the oxidative pretreatment of lignocellulose while protecting hydrolytic enzymes from oxidative damage. Interestingly, a similar sequential induction of oxidative and hydrolytic mechanisms was observed by Navarro et al. [75], who showed that AA9 LPMO, which cleave cellulose by oxidation, were produced ahead of hydrolytic cellobiohydrolases when soil-inhabiting fungus *Laetisaria arvalis* was grown on crystalline cellulose.

Several studies also highlighted differences in gene regulation when the fungi were grown on different woody substrates. For example, Daly et al. [76] showed that white-rot fungus *Dichomitus squalens* produced more mannolytic enzymes during growth on softwood (which has a higher mannan content) and more xylanolytic enzymes during growth on hardwood (which has a higher xylan content). In addition, MnP were more abundantly produced during growth on softwood, which contains guaiacyl (G) lignin, than on hardwood, which contains syringyl/guaiacyl (S/G) lignin. These findings were in line with the acquisition of MnP by white-rot fungi for adaptation to gymnosperm woods [31], and with MnP having a higher efficiency for the modification of G-lignin compared to S-lignin [77].

The role of gene-expression regulation in the adaptive response of the fungi to lignocellulosic substrates was further evidenced by comparative analysis of the four *Pycnoporus* species described to date [78]. Despite the species coming from geographically distant areas, the sequenced genomes did not show any major rearrangements in their structure, and showed strong conservation in the CAZome compositions. However, the species exhibited different abilities to grow on cellulose, pine wood, or aspen wood in vitro, indicating that the information obtained from genome sequences and CAZome portfolios was not sufficient to predict the ability of a strain to grow on recalcitrant raw biomass. Nevertheless, comparative analysis of the transcriptomes and secretomes of the fungi facilitated the identification of a conserved set of CAZymes for cellulose and hemicellulose breakdown. On the other hand, the sets of enzymes involved in lignin breakdown and in the detoxification of oxidative products released during lignocellulose degradation differed among species.

## 6. Conclusions

Altogether, recent findings and ongoing developments highlighted fungi as pivotal actors in the balance of CO_2_ storage and CO_2_ emissions in natural environments, and as powerful toolkits for the development of biotechnologies centered on renewable carbon. 

In laboratory conditions, different fungal species show different abilities to deconstruct the lignocellulose polymers. This functional diversity indicates the diversity of enzyme sets that can be investigated to optimize the valorization of plant biomass of diverse origins and compositions. This fungal biodiversity also offers the possibility to screen for optimal enzymatic systems aimed at particular biomass valorization routes (e.g., the selective degradation of lignin with the preservation of saccharides for the production of bioenergy).

In some examples, however, this functional diversity was related to differences in gene expression profiles, demonstrating that the gene repertoires identified from genome sequences are not sufficient to predict the ability of a strain to grow on recalcitrant raw biomass, and functional analyses are necessary to understand the enzymatic systems at play during lignocellulose degradation. However, analysis of sets of CAZymes commonly mobilized by fungi during growth on lignocellulosic substrates offers the possibility to identify key enzymatic mechanisms that unlock plant-biomass recalcitrance.

## Figures and Tables

**Table 1:** Fungal carbohydrate-active enzymes (CAZymes) active on plant-cell walls. Activities were reported in Henriksson et al., Cantarel et al., Gruber and Seidl-Seibothl, Floudas et al., Sipos et al., Couturier et al., Andlar et al., and Filiatrault-Chastel et al. [16,17,18,19,20,21,22,23].

Substrate	Enzyme Classes	Enzymatic Activity	CAZy Family
Lignin	copper radical oxidase	Laccase	AA1_1
	Class II heme peroxidases	Manganese peroxidase	AA2
		Lignin peroxidase	
		Versatile peroxidase	
			
Hemicellulose	Glycoside Hydrolases	Endo-1,4-β-xylanase	GH5_22, GH8, GH10, GH11, GH30_7
		xyloglucanase	GH74, GH44
		Endo-β-1,4-mannanase	GH5_1, GH5_7, GH26, GH113, GH134
		Endo-α-1,5-arabinanase	GH93
		β-1,3-glucanase	GH16
		α-L-arabinofuranosidase	GH43, GH51, GH54, GH62
		β-Glucuronidase	GH115, GH2
		α-1,2-glucuronidase	GH67, GH115
		β-mannosidase	GH2, GH5_2
		β-Galactosidase	GH35, GH53
		α-Galactosidase	GH27, GH36
		β-xylosidase	GH52, GH54, GH120, GH30_1, GH39
		α-L-fucosidase	GH29, GH95, GH141
	Carbohydrate Esterases	Acetylxylan esterase	CE1, CE2, CE3, CE4, CE6, CE16
		Cutinase	CE5
		Glucuronyl methyl esterase	CE15
	Auxiliary activities	Lytic polysaccharide monooxygenase	AA14
			
Cellulose	Glycoside Hydrolases	Endoglucanase	GH5,4, GH5_5, GH12, GH45,GH74, GH131
		cellobiohydrolase	GH6, GH7, GH5_1, GH48
		β-Glucosidase	GH1, GH3, GH30_1, GH5_7, GH5_22
	Auxiliary activities	Lytic polysaccharide monooxygenase	AA9, AA16
			
Pectin	Glycoside Hydrolases	Polygalacturonases	GH28, GH78
		β-glucuronyl hydrolase	GH88, GH105
		α-L-rhamnosidase	GH78, GH106
		β-1,4-galactanase	GH53
	Polysaccharide Lyases	Polygalacturonate lyase	PL1, PL3, PL9
		Rhamnogalacturonan lyas	PL4, PL11, PL26
	Carbohydrate Esterases	Rhamnogalacturonan acetylesterase	CE12
		Pectin methylesterase	CE8
			
Cutin	Carbohydrate Esterases	Cutinase	CE5
			
	Auxiliary activities	FAD-dependent (GMC) oxidoreductase	AA3
		vanillin alcohol oxidase	AA4
		copper radical oxidase	AA5
		benzoquinon reductase	AA6
		glycooligosaccharide oxidase	AA7
		pyrroloquinoline quinone-dependent oxidoreductase	AA12
		Cellobiose dehydrogenase	AA8-AA3
			
	Carbohydrate binding modules	Xylan, galactan	CBM13
		cellulose	CBM1, CBM63
		pectin	CBM67
